# Prostate cancer and the human papilloma virus: causative association, role of vaccines, and the impact of the COVID-19 pandemic

**DOI:** 10.1038/s41391-021-00404-6

**Published:** 2021-06-18

**Authors:** Naomi Morka, Joseph M. Norris, Mark Emberton, Daniel Kelly

**Affiliations:** 1 University College London Medical School, London, UK; 2 UCL Division of Surgery & Interventional Science, University College London, London, UK; 3 Department of Urology, University College London Hospitals NHS Foundation Trust, London, UK; 4 School of Healthcare Sciences, Cardiff University, Cardiff, Wales UK

**Keywords:** Cancer, Urological cancer, Prostate cancer

## Abstract

Prostate cancer affects a significant proportion of men worldwide. Evidence from genetic and clinical studies suggests that there may be a causal association between prostate cancer and the human papilloma virus (HPV). As HPV is a vaccine-preventable pathogen, the possibility of a role in prostate cancer causation may reinforce the importance of effective HPV vaccination campaigns. This is of particular relevance in light of the COVID-19 pandemic, which may have considerable effects on HPV vaccine uptake and distribution.

Prostate cancer is the commonest solid-organ malignancy in men worldwide and represents a significant healthcare burden. Although the feasibility of modifying recognised risk factors for prostate cancer is currently low, there is evidence to suggest that the vaccine-preventable pathogen, Human Papilloma Virus (HPV) may be associated with the pathogenesis of prostate cancer [1]. HPV is most notably known for its ability to cause cervical cancer in women, but it is also associated with anogenital and oral cancers [2]. There are multiple subtypes of HPV and these can be classified according whether they are high or low-risk for the development of cancer [3]. Recent work has identified the presence of high-risk HPV in prostate tumours and has led to speculation that there may be a causal relationship between HPV exposure and the development or promotion of cancer in the prostate [2–6]. It is therefore important to explore, or indeed question this possibility, especially given the established efficacy of HPV vaccination and the negative effects of the coronavirus disease 2019 (COVID-19) pandemic on HPV vaccine delivery [7].

The potential role of HPV in the causation of prostate cancer has been the subject of considerable debate and the focus of several recent systematic reviews (Table 1). HPV is one of several microorganisms found in the prostate gland, and has been identified in both benign and cancerous samples [4]. Additionally, studies suggest that the prevalence of high-risk HPV in benign prostatic tumours is comparable to those found in the presence of prostate cancer [3], and as such, it could be argued that the presence of HPV may be coincidental. However, a recent systematic review of 26 studies by Lawson and Glenn [4] concluded that there was an increased proportion of high-risk HPV types in prostate cancer compared to benign and healthy tissue. This is supported by results from a recent meta-analysis which suggested that HPV infection was associated with an increased likelihood of prostate cancer development, with an odds ratio of 2.27 (95% confidence interval, 1.40–3.69) [5]. The apparent discrepancy in findings across studies may be attributed to differing experimental techniques used to confirm the presence of HPV infection. Moreover, the suitability of serology to identify HPV has been questioned, as it may not provide a true assessment of HPV infection within the prostate itself [4]. Also, there is a variation in the prevalence and distribution of HPV subtypes globally, and it is possible that this may have been a contributing factor to the observed heterogeneity of findings in case–control studies [4, 5]. Indeed, assessing a person’s degree of exposure to HPV may not provide sufficient foundation to infer effect from this pathogen within the prostate.

**Table 1:** Recent systematic reviews and meta-analyses examining the association between prostate cancer and HPV.

Review author, Ref.	No. included studies	Key findings
Russo et al. [2]	30	The odds of developing prostate cancer were increased in men positive for HPV-16 (pooled OR = 1.37, *p* < 0.01), but not in HPV-18 positive men.
Lawson et al. [4]	26	High-risk HPVs were identified in 22.6% of prostate cancers, compared to 8.6% of benign or normal prostate tissues (*p* = 0.001).
Yin et al. [5]	24	In HPV-positive men, the pooled OR for developing prostate cancer was 2.27 (95% CI = 1.40–3.69).
Moghoofei et al. [6]	24	There was a positive association between HPV and prostate cancer (OR = 1.281, *p* = 0.026) and a higher incidence of HPV-16 in prostate tumours.

*No.* Number, *HPV* human papilloma virus, *OR* odds ratio, *p* probability, *CI* confidence interval.

However, at the genetic level, there appears to be variation in the nature of HPV infection between benign and cancerous tumours. A study by Glenn et al. [3] demonstrated a reduction in the expression of the E6 and E7 HPV oncogenes during the progression of prostate tumours from benign to malignant states. Using RNA sequencing, the same study found that HPV was likely present in an activated state in a proportion of prostate cancers. This evidence raises the possibility that HPV could be an early pathogenic contributor to prostate cancer development, but may have a diminished role in later stages [4]. This is substantiated by the observation that koilocytes are present within some prostate tumours [8]. These cells are indicative of HPV infection and are known to signify the early stages of oncogenesis in cervical cancer [4]. Of note, the possible role of HPV in prostate cancer causation may differ from its mechanism of action in cervical carcinogenesis [4]. In a population of immunocompromised patients, there was a greater incidence in a variety of cancers, including cervical malignancies, but the risk of prostate cancer was not increased [9]. While some maintain that this evidence reduces the likelihood of a role for HPV in prostate cancer causation, it may be argued instead that these findings highlight the possibility of an indirect mechanism of oncogenesis [4, 9]. HPV may lead to mutations in *APOBEC3B*, a gene which has been found to have a protective role against the effects of viruses [10]. Mutant versions of this gene have been observed in prostatic tumours [11]. In addition, HPV may play a role in apoptotic, inflammatory and angiogenic processes within prostatic tissue, all of which are key elements of carcinogenesis [1].

Due to the possible role of HPV in prostate cancer causation, it may be argued that it is increasingly important to intensify current HPV vaccination strategies, with a sustained gender-neutral approach. This may be of greater relevance than ever before, as the COVID-19 pandemic has posed a significant challenge to HPV vaccination campaigns. Unfortunately, short-term measures to mitigate the spread of COVID-19, such as school closures and limited clinic visits, have led to a fall in HPV vaccination rates [12]. In the United States, there was an 80% reduction in HPV vaccine orders by the end of March 2020, compared to the same time in the previous year [13, 14] and a parallel marked reduction of HPV vaccine uptake in Europe [7]. It is unclear what the long-term effects of this situation will be, but there has been speculation that the increased vaccine hesitancy seen during the COVID-19 pandemic could negatively impact future HPV vaccination campaigns [14]. However, some maintain that the successful results of COVID-19 vaccination efforts could increase public confidence in vaccines, leading to greater uptake of the HPV vaccine [7, 14]. Nonetheless, there is a need to support the expeditious revivification of HPV vaccination campaigns, redesigning them where necessary, to suit the current circumstances.

Despite the incongruity of evidence surrounding HPV exposure and prostate cancer causation, extant evidence suggests an association worthy of further investigation. The significance of a confirmed role of HPV in the development of prostate cancer is weighty, and would strengthen calls for current generic vaccination programmes [7], ultimately, providing greater protection for men at risk, for whom modifiable risk factors have, as yet, been elusive.
